# Microbiological and functional traits of peri-implant mucositis and correlation with disease severity

**DOI:** 10.1128/msphere.00059-24

**Published:** 2024-07-09

**Authors:** Ziying Feng, Jinzan Zhu, Limin Zhang, Chunchun Li, Duyao Su, Huihui Wang, Youcheng Yu, Liang Song

**Affiliations:** 1 Department of Stomatology, Shanghai Fifth People’s Hospital, Fudan University, Shanghai, China; 2 Department of Stomatology, Zhongshan Hospital of Fudan University, Shanghai, China; University of Michigan-Ann Arbor, Ann Arbor, Michigan, USA

**Keywords:** dental implant, metagenomics, whole-genome sequencing, dysbiosis, sulcus bleeding index

## Abstract

Osseointegrated dental implants replace missing teeth and create an artificial surface for biofilms of complex microbial communities to grow. These biofilms on implants and dental surfaces can trigger infection and inflammation in the surrounding tissue. This study investigated the microbial characteristics of peri-implant mucositis (PM) and explored the correlation between microbial ecological imbalance, community function, and disease severity by comparing the submucosal microflora from PM with those of healthy inter-subject implants and intra-subject gingivitis (G) within a group of 32 individuals. We analyzed submucosal plaques from PM, healthy implant (HI), and G sites using metagenome shotgun sequencing. The bacterial diversity of HIs was higher than that of PM, according to the Simpson index. Beta diversity revealed differences in taxonomic and functional compositions across the groups. Linear discriminant analysis of the effect size identified 15 genera and 37 species as biomarkers that distinguished PM from HIs. Pathways involving cell motility and protein processing in the endoplasmic reticulum were upregulated in PM, while pathways related to the metabolism of cofactors and vitamins were downregulated. Microbial dysbiosis correlated positively with the severity of clinical inflammation measured by the sulcus bleeding index (SBI) in PM. *Prevotella* and protein processing in the endoplasmic reticulum also correlated positively with the SBI. Our study revealed PM’s microbiological and functional traits and suggested the importance of certain functions in disease severity.

**IMPORTANCE:**

Peri-implant mucositis is an early stage in the progression of peri-implantitis. The high prevalence of it has been a threat to the widespread use of implant prosthodontics. The link between the submucosal microbiome and peri-implant mucositis was demonstrated previously. Nevertheless, the taxonomic and functional composition of the peri-implant mucositis microbiome remains controversial. In this study, we comprehensively characterize the microbial signature of peri-implant mucositis and for the first time, we investigate the correlations between microbial dysbiosis, functional potential, and disease severity. With the help of metagenomic sequencing, we find the positive correlations between microbial dysbiosis, genus *Prevotella*, pathway of protein processing in the endoplasmic reticulum, and more severe mucosal bleeding in the peri-implant mucositis. Our studies offer insight into the pathogenesis of peri-implant mucositis by providing information on the relationships between community function and disease severity.

## INTRODUCTION

With the emergence of dental implants as a prosthodontic treatment option, peri-implant infections have become a byproduct of progress in bioengineering technology (1). Based on the extent of inflammation, peri-implant infections can be categorized into peri-implant mucositis (PM), which is limited to soft tissue and peri-implantitis (PI), which involves the surrounding bone (2). PM is considered reversible and a precursor of PI (3). PM’s prevalence varies from 19% to 65% depending on the case definition (4, 5). The diagnostic criteria for peri-implant infections mainly depend on clinical and radiographic examinations. Therefore, the clinical assessment of bleeding signs is essential for detecting inflammation around the implant.

Plaque accumulation is a major etiological factor of PM, as proven in animal and human experimental studies (6). Various approaches have been adopted to analyze the composition of peri-implant plaques over the years. Previous studies have relied on anaerobic culture-based techniques and phase contrast microscopy (7). Polymerase chain reaction, fluorescence *in situ* hybridization, and DNA-DNA checkerboard hybridization provided a clearer outline of peri-implant infection-related bacteria. However, these closed-ended molecular techniques require preselection of primers and can only target bacteria of specific taxa. This leads to selection bias because the targeted taxa are usually based on known periodontal pathogens (1). Currently, high-throughput DNA sequencing technologies enable the comprehensive community profiling of peri-implant plaques. More functional descriptions of microbial communities can be obtained using whole-genome sequencing (8), which reconstructs a complete bacterial metagenome *via* shotgun metagenome data and predicts the functional genetic potential within the community (9).

In recent years, the peri-implant microbial community characteristics of PI and healthy implant (HI) conditions have received more attention. PM’s microbiome is controversial as an intermediate stage. One study showed a similarity in the microbial profiles of PM and HIs (10), while another suggested that PM’s microbiome features were similar to those in PI (11). Changes in the microbial community have been observed in PM (12–15); nevertheless, further studies are necessary to gain deeper insight into the structure of PM’s microbiome. Considering the similar clinical manifestations of peri-implant and periodontal infections, comparisons have revealed differences in microbial aspects between PM and gingivitis (G) (16–18). Moreover, correlations between inflammation and the microbiome have been explored, and it has been noticed that different grades of bleeding on probing correspond to different submucosal microbiomes in PM in a cohort of patients with periodontitis (19). In a recent study, microbial dysbiosis in mucosal plaques was not related to the probing depth or bleeding on probing in PM; however, marginal bone loss was positively correlated with microbial dysbiosis in PI (11). It was also shown that PM sites with suppuration harbored a more imbalanced microbial community with more pathogenic taxa compared with PM sites without suppuration (20). However, the relationships between taxonomic and functional biomarkers and clinical inflammatory conditions have not been determined.

This study aimed to characterize the microbial signature of PM by comparing it with that of healthy inter-subject implants and intra-subject G. Both taxonomic and functional compositions of the microbiome were investigated using metagenomic shotgun sequencing. In addition, we explored the correlations between microbial dysbiosis, community function, and disease severity.

## RESULTS

### Clinical characteristics and overview of microbiome profile

Here, we studied the submucosal microbiome associated with PM in a cohort of partially edentulous Chinese residents in Shanghai. We included 32 subjects aged 27–77 years with at least one dental implant diagnosed as PM (*n* = 20), control subjects with at least one clinically HI (*n* = 12), and a natural tooth adjacent to the PM sites from subjects with G (*n* = 10).

No significant differences exist in age, sex, presence of periodontitis, smoking habits, oral hygiene practices, or implant location between PM and HI. The participants in the G group were selected from the PM group. Details regarding the clinical examination of each study site are presented in Table S1. Based on the analysis of clinical data from the sampled population, the HI sites showed lower PLI and SBI values than the PM sites (Table 1).

**TABLE 1:** Demographic and clinical features of the study cohort^*a*^

	PM (*n* = 20)	HI (*n* = 12)	*P*-value
Patient age (years, mean ± SD)	43.70 ± 12.40	50.54 ± 13.49	0.20
Gender (M/F)	15/5	6/6	0.15
Periodontitis (Y/N)	5/15	0/12	0.06
Smoking (Y/N)	4/16	1/11	0.38
Frequency of oral hygiene per day (mean ± SD)	2.00 ± 0.00	2.10 ± 0.73	0.17
Application of dental floss (Y/N)	9/11	5/7	0.85
Location of the sampled site			
Maxillary/mandibular	8/12	2/10	0.17
Anterior/ posterior	0/20	0/12	-
PLI (mean ± SD)	2.05 ± 0.38	1.36 ± 0.65	0.008^*b*^
PPD (in mm, mean ± SD)	3.35 ± 0.79	2.91 ± 0.67	0.15
SBI (mean ± SD)	2.15 ± 0.85	0.00 ± 0.00	<0.001^*b*^
SUP (Y/N)	2/18	0/12	0.39
MBL (in mm, mean ± SD)	0.93 ± 0.43	0.80 ± 0.29	0.26

^*a*^ PM: peri-implant mucositis; HI: clinically healthy implant. M: male; F: female. Y: yes; N: no. PLI: plaque index; PPD: peri-implant/tooth pocket probing depth; SBI: sulcus bleeding index; SUP: suppuration; MBL: marginal bone loss on radiography.

^*b*^ Statistically significant.

A total of 42 submucosal/subgingival microbial plaque samples were collected for metagenomic analysis by whole-genome shotgun sequencing on the Illumina NovaSeq platform. After quality filtering and removal of non-target reads, approximately 430 Gb of metagenomic reads with an average of 10.24 ± 0.77 Gb (mean ± standard deviation) per sample were obtained. The species accumulation curve of each study group (Fig. S1) demonstrated that the sample size is sufficient for the microbiological analysis in this study.

**Fig 1:**
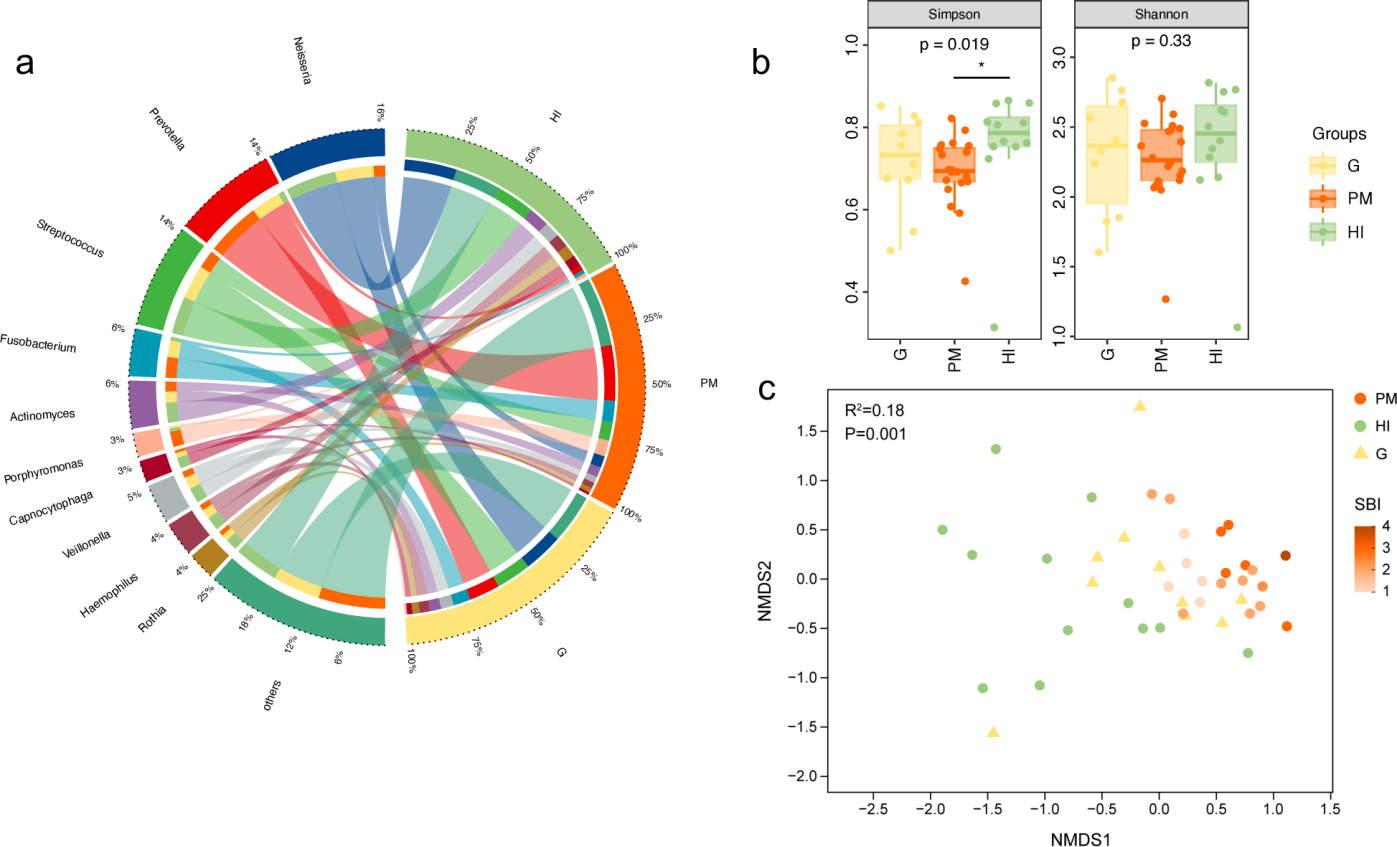
(**a**) Taxonomic composition at genus-level correspondence across the three study groups. (**b**) Alpha-diversity distributions show lower levels in PM compared with HIs. The *P*-values were obtained using the Kruskal–Wallis rank sum test. (**c**) NMDS plot based on the Bray–Curtis distances reveals a clear distinction in clustering between the groups. Colors intensity of bars reflects the corresponding SBI score: the darker the color, the higher the SBI score. SBI, sulcus bleeding index; NMDS, nonmetric multidimensional scaling.

To evaluate the microbial composition of the three study groups, we mapped the metagenomic reads to reference genomes with species-level abundance. The six dominant phyla in all the samples were *Bacteroidetes, Proteobacteria, Firmicutes, Actinobacteria, Fusobacteria,* and *Spirochaetes*, accounting for over 97.2% of the total assigned taxa. The 10 dominant genera were *Prevotella, Neisseria, Streptococcus, Fusobacterium, Actinomyces, Veillonella, Haemophilus, Porphyromonas, Rothia,* and *Treponema*. The results of taxonomic assignment at the genus and phylum levels are presented in Fig. 1a; Fig. S2, respectively.

The submucosal plaque microbiome from the HI sites showed significantly greater alpha diversity than that from the PM sites in the Simpson index (*P* < 0.05, Kruskal–Wallis test); however, significance was not achieved in the Shannon index. Meanwhile, the subgingival microbiome from G had a lower Simpson and Shannon diversity index than that of the HI, although neither of the differences reached statistical significance (Fig. 1b). To further compare the variation in microbial compositions by different groupings, beta diversity analysis based on the Bray-Curtis distance was performed and visualized using the NMDS ordinations. The discriminating microbial community structures among the study groups are shown in Fig. 1c (PERMANOVA, R^2^ = 0.18, *P* = 0.001). When the PM samples were classified by their corresponding SBI, the microbial profiles of the samples with lower SBI were similar to those of the HI samples.

### Microbial signatures of peri-implant mucositis

**Fig 2:**
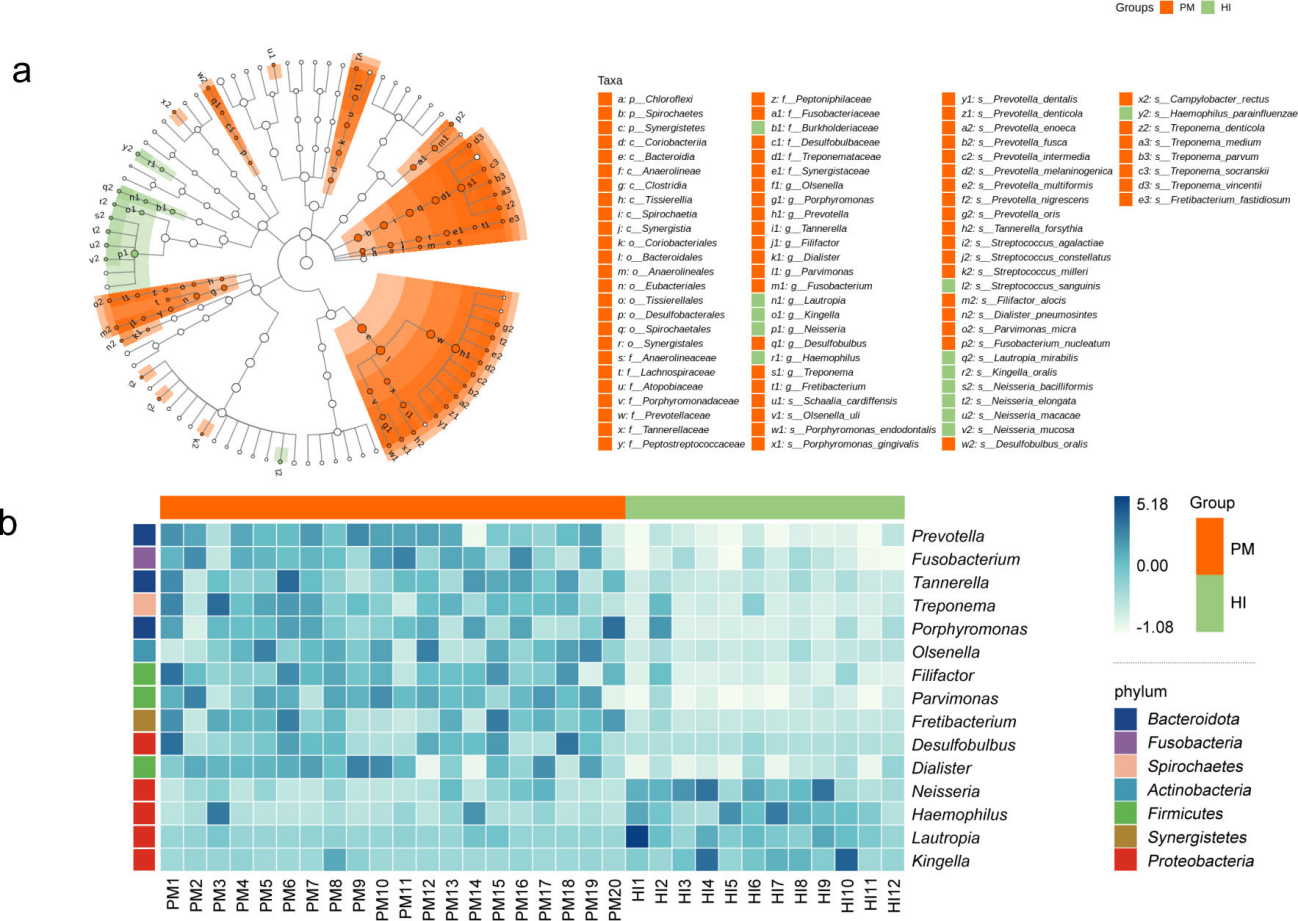
(**a**) LEfSe analysis identifies biomarkers between the PM and HI groups. The cladogram plots show discriminating taxa at different levels of taxonomic classification (phylum, class, order, family, genus, and species). An LDA value of ≥3.0 is the inclusion threshold. (**b**) Differential abundance patterns of LEfSe biomarkers between PM and HIs. The heatmap shows relative abundances (normalized z-scores) of discriminating genera; darker color indicates high abundance and lighter shades indicate low abundance. HI, healthy implant; LDA; linear discriminant analysis; LEfSe, linear discriminant analysis effect size; PM, peri-implant mucositis.

As shown in the NMDS plot (Fig. 1c), most samples from the PM and HI groups formed two clusters, indicating differences in beta diversity between the two groups, which was further confirmed using ANOSIM (PM/HI: R = 0.61 and *P* = 0.001; PM/G: R = 0.32, *P* = 0.002). As the distribution on the genera level in 32 samples from PM and HI is shown in Fig. 1a, the PM submucosal bacterial communities were dominated by representatives of *Prevotella* (25.2%), *Fusobacterium* (9.5%), *Streptococcus* (8.4%), *Porphyromonas* (7.2%), and *Treponema* (5.4%), while the HI submucosal bacterial communities were dominated by representatives of *Neisseria* (23.5%), *Streptococcus* (17.3%), *Actinomyces* (9.3%), *Veillonella* (6.9%), and *Haemophilus* (6.2%). Differential abundance analyses at various levels between the groups were performed using LEfSe analysis. We identified discriminatory taxa in 15 genera and 37 species (Fig. 2a; Fig. S3a). A heatmap of these 15 differential genera is shown in Fig. 2b. Based on these genera, the MDI of PM was calculated to evaluate microbial dysbiosis. The formula used is as follows:


$$MDI=log\left(\frac{\left[total\;abundance\;of\;taxa\;increased\;in\;PM\right]}{\left[total\;abundance\;of\;taxa\;decreased\;in\;PM\right]}\right)$$

**Fig 3:**
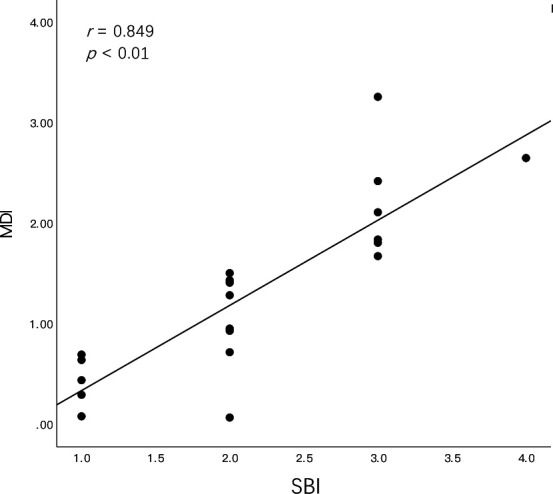
Correlation between the microbial dysbiosis index (MDI) of submucosal microbiome and the SBI in the PM group. SBI, sulcus bleeding index.

The results showed that the MDI was positively correlated with the mean SBI in the PM group (R = 0.849, *P* < 0.01; Fig. 3). *Fusobacterium nucleatum, Prevotella intermedia, Prevotella nigrescens, Prevotella oris, Prevotella melaninogenica, Prevotella denticola, Prevotella multiformis, Porphyromonas gingivalis, Porphyromonas endodontalis, Tannerella forsythia, Treponema socranskii, Treponema denticola, Filifactor alocis,* and *Parvimonas micra* were among the most abundant species in PM and were significantly more abundant than those in HIs (Fig. S4).

**Fig 4:**
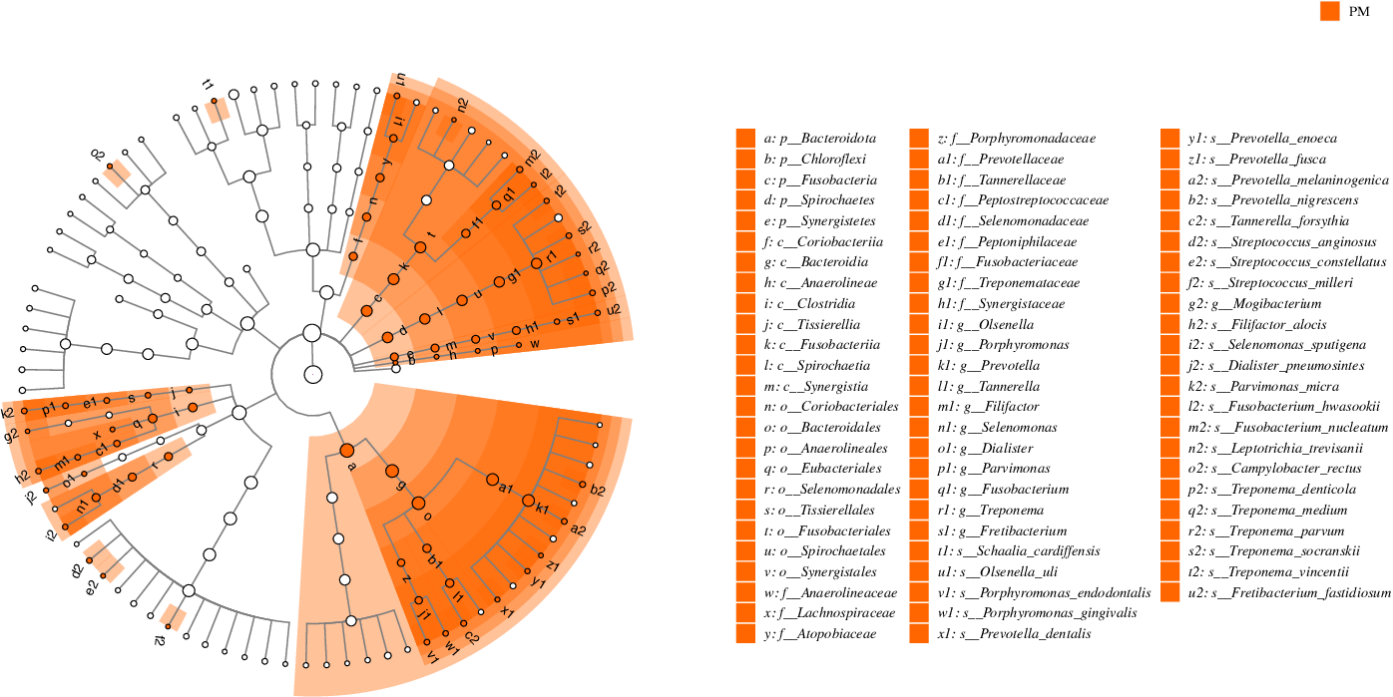
Biomarkers indicated by LEfSe analysis between the PM and G groups. The cladogram plots show discriminating taxa at different levels of taxonomic classification (phylum, class, order, family, genus, and species). An LDA value of ≥3.0 is the inclusion threshold. LDA, linear discriminant analysis; LEfSe, linear discriminant analysis effect size.

The subgingival bacterial communities in G were dominated by *Neisseria* (17.9%), *Streptococcus* (15.9%), *Prevotella* (13.9%), *Fusobacterium* (7.7%), and *Veillonella* (5.7%). Differential abundance analyses at various levels between the groups were performed using LEfSe analysis. We identified discriminatory taxa in 11 genera and 28 species (Fig. 4; Fig.S3b).

Although the microbiomes from both the PM and G sites contained high proportions of *F. nucleatum, P. nigrescens,* and *P. micra* (Fig. S4)*,* their relative abundances still varied between groups, showing enrichment in PM. However, the most frequently occurring species, such as *P. oris, P. intermedia, P. denticola, Veillonella párvula,* and *Haemophilus parainfluenzae,* were not significantly different in their relative abundances between PM and G.

### Functional biomarkers of PM

**Fig 5:**
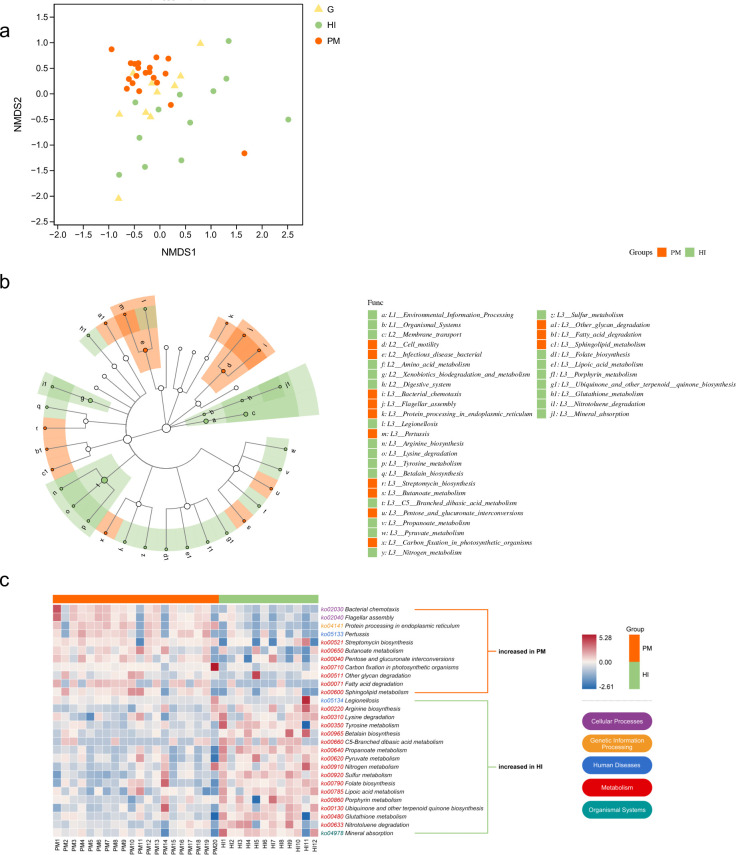
(**a**) NMDS plot based on Bray–Curtis distances indicates distinct functional profiles across the three categories. (**b**) LEfSe analysis identifies biomarkers for the PM group versus the HI group. The cladogram plots show discriminating functional units at different levels of KEGG. An LDA value of ≥3.0 is the inclusion threshold. (**c**) Heatmap shows relative abundances (normalized z-scores) of LEfSe-identified KEGG pathways discriminating the PM and HI groups; red indicates high abundance, blue indicates low abundance; the color of ko numbers corresponds to their KEGG level 1 classification. HI, healthy implant; KEGG, Kyoto Encyclopedia of Genes and Genomes; LDA, linear discriminant analysis; LEfSe, linear discriminant analysis effect size; NMDS, nonmetric multidimensional scaling; PM, peri-implant mucositis.

**Fig 6:**
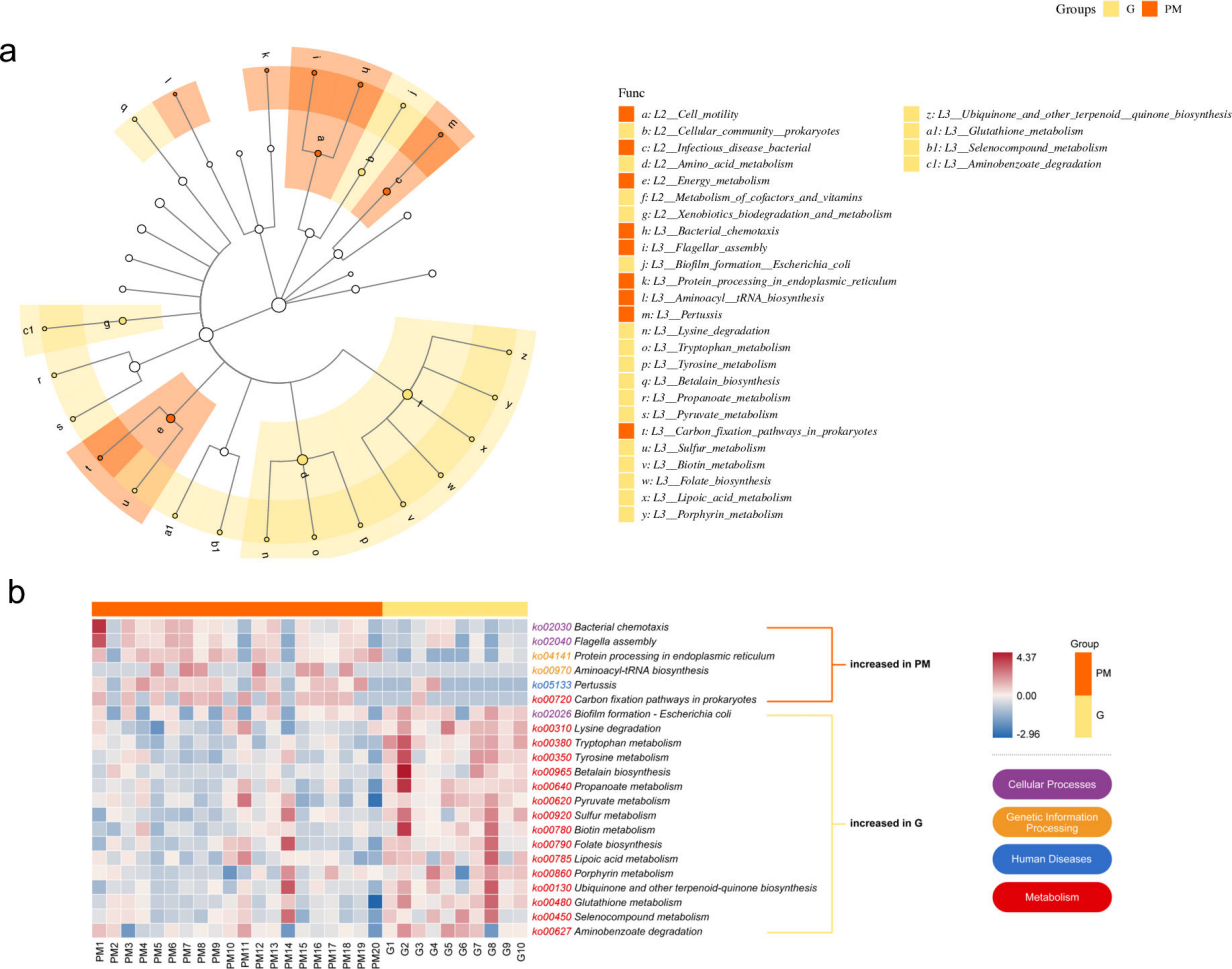
(**a**) LEfSe analysis identifies biomarkers differentiating the PM and G groups. The cladogram plots show discriminating functional units at different levels of KEGG. An LDA value of ≥3.0 is the inclusion threshold. (**b**) Heatmap shows relative abundances (normalized z-scores) of LEfSe-identified KEGG pathways discriminating the PM and G groups; red indicates high abundance, blue indicates low abundance; the color of ko numbers corresponds to their KEGG level 1 classification. G, gingivitis; KEGG, Kyoto Encyclopedia of Genes and Genomes; LDA, linear discriminant analysis; LEfSe, linear discriminant analysis effect size; PM, peri-implant mucositis.

Functional annotations of the genes were performed using the KEGG database, the overall profile of which is presented in an NMDS plot (PERMANOVA, R^2^ = 0.11, *P* = 0.001, Fig. 5a). The functional profiles of the three groups were distinguished and confirmed by ANOSIM (PM/HI: R = 0.59, *P* = 0.001; PM/G: R = 0.36, *P* = 0.002). Differential abundance analyses at various levels between the groups were performed using LEfSe analysis. Setting a threshold of 3 in the LDA score, 28 pathways were found to be discriminative at KEGG level 3 between PM and HIs, 11 of which, including bacterial chemotaxis, flagellar assembly, protein processing in the endoplasmic reticulum (ER), streptomycin biosynthesis, and fatty acid degradation, were enriched in PM, while the other 17 were enriched in HIs (Fig. 5b). A heatmap including the 27 differential pathways is shown in Fig. 5c. Differences between PM and G were observed in 22 pathways, with 6 of them enriched in PM, namely, bacterial chemotaxis, flagellar assembly, protein processing in the ER, aminoacyl rRNA biosynthesis, pertussis, and carbon fixation pathways in prokaryotes (Fig. 6a). Pathways of carbon fixation in photosynthetic organisms and thiamine metabolism were shared by PM and G, with high abundance and no significant differences between the groups. A heatmap including the 22 differential pathways between PM and G is shown in Fig. 6b.

### Correlations of clinical features with taxa and function units

**Fig 7:**
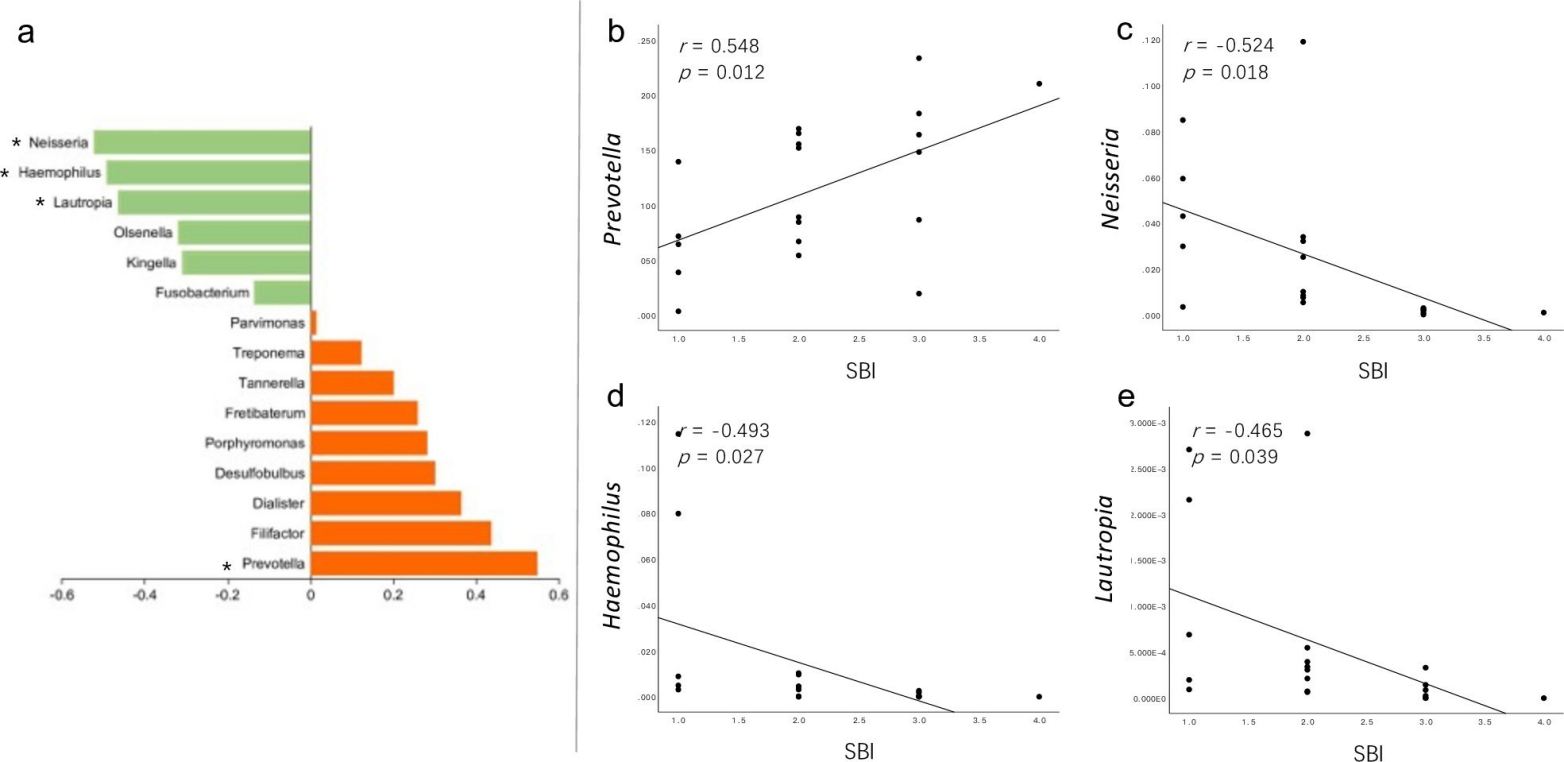
(**a**) Relationship between the SBI in patients with PM and 15 differentially abundant genera identified in LEfSe analysis when comparing the PM with HIs, based on Spearman correlation analysis. Orange and green colors indicate positive and negative correlations, respectively, with the column lengths indicating the strength of Spearman’s correlation coefficient. Significant correlations (*P* < 0.05) are marked with asterisks. Specific genus-level correlation analyses are presented in panels (b–e). HI, healthy implant; LEfSe, linear discriminant analysis effect size; SBI, sulcus bleeding index; PM, peri-implant mucositis.

**Fig 8:**
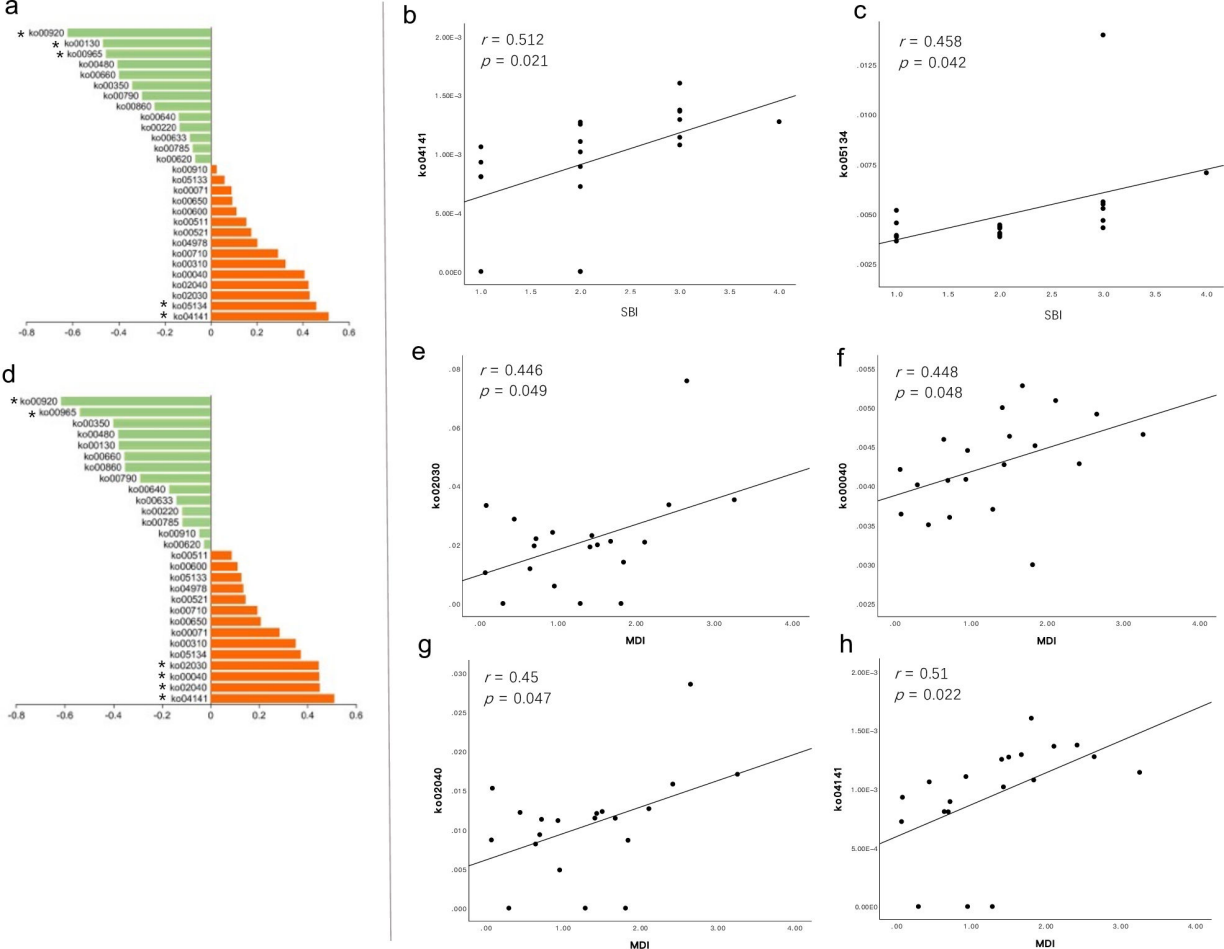
(**a**) Spearman correlation analysis reveals relationships between the SBI in PM and differentially abundant KEGG pathways, identified by LEfSe analysis (PM versus HI). Orange and green colors indicate positive and negative correlations, respectively, and the lengths of the columns represent Spearman’s correlation coefficients. Significant correlations (*P* < 0.05) are marked with asterisks, and the correlation analysis is presented (**b and C**). (**d**) Similar analysis for the MDI versus differentially abundant KEGG pathways (PM versus HIs). Orange and green colors indicate positive and negative correlations, respectively, and the lengths of the columns represent Spearman’s correlation coefficients. Significant correlations (*P* < 0 0.05) are marked with asterisks, and the correlation analysis is presented in panels (e–h). HI, healthy implant; KEGG, Kyoto Encyclopedia of Genes and Genomes; LEfSe, linear discriminant analysis effect size; MDI, microbial dysbiosis index; SBI, sulcus bleeding index; PM, peri-implant mucositis.

We further explored the relationships between several elements in the PM samples. Among the 15 discriminative genera observed between PM and HIs (Fig. 7a), 11 increased in PM, and the correlation between their relative abundances and the SBI revealed that only *Prevotella* had a significant positive correlation (R = 0.548, *P* = 0.012, Fig. 7b) in its abundance with the SBI in PM. However, the relative abundances of the decreasing genera *Neisseria*, *Haemophilus*, and *Lautropia* were negatively correlated with the SBI (Fig. 7c through e). Referring to the relationship between KEGG pathways enriched in PM and the SBI (Fig. 8a), only ko04141 (protein processing in ER) and ko05134 (legionellosis) showed a significant correlation with the SBI (R = 0.512, *P* = 0.021, Fig. 8b; R = 0.458, *P* = 0.042, Fig. 8c), and together with three other pathways (Fig. 8d), ko02030 (R = 0.446, *P* = 0.049, Fig. 8e), ko00040 (R = 0.448, *P* = 0.048, Fig. 8f), ko02040 (R = 0.450, *P* = 0.047, Fig. 8g), and ko04141 (R = 0.510, *P* = 0.022, Fig. 8h) was also significantly associated with the MDI.

## DISCUSSION

PM is a common implant prosthodontics-associated disease. As the precursor of PI, it is reversible and needs to be treated cautiously. Correlations between the submucosal plaque microbiota and the pathogenesis of PM have been analyzed by previous studies based on DNA-DNA Checkerboard hybridization or 16s rRNA sequencing techniques (10–13, 15). In this study, by comparing the submucosal microbiota of PM with inter-subject HI and intra-subject G using metagenomic shotgun sequencing, we aim to profile the microbial signature of PM and explore the relationships between taxonomic features, microbial dysbiosis, community function, and disease severity. To the best of our knowledge, this is the first study to explore the correlation between the functional potential of the plaque microbiome and the severity of soft tissue lesions by different degrees of mucosal bleeding in PM. We found that certain functions correlated with microbial dysbiosis and more severe mucosal bleeding in the PM. These findings provide a better understanding of the onset of peri-implant biofilm-related infections.

The submucosal microbiome signature in PM remains controversial. The alpha diversity in the submucosal microbial communities at PM sites can be similar to that at HI sites, as a recent study showed no significant difference in the alpha diversity of the microbial community in terms of the Shannon index (19). However, other studies have reported significantly lower alpha diversity in PM (14, 21) and higher alpha diversity in diseased implants in the non-smoking cohorts (12). In this study, we found no significant difference in alpha diversity between PM and HI according to the Shannon index; however, a significant difference was observed in the Simpson index. Hence, the Shannon and Simpson indices are classic methods for measuring community diversity (22). The advantage of using the Simpson diversity index is that it provides more information about species abundance rather than simply the number of species present, and it accounts for both low and common abundant species (23). Consistent with previous studies (14, 24, 25), we found significant differences in the structures of the submucosal microbial communities between the PM and HI sites. The beta diversity of PM formed a separate cluster from that of HIs in the NMDS plots. By contrast, another study observed that PM’s microbiome structure was similar to that of HIs (10). Notably, when we labeled the PM samples according to the corresponding SBI on the NMDS plot, the samples with a smaller SBI were closer to the HI samples than their higher SBI counterparts (Fig. 1c), indicating a more similar microbial community structure to HIs when the clinical inflammatory status was lower.

PM is considered a precursor of PI; therefore, the dominant flora in PM includes a proportion of the dominant flora in PI. In our study, the taxonomic compositions of the peri-implant submucosal plaques were analyzed at various levels, and the most abundant phyla in the peri-implant niches comprised *Bacteroides*, *Proteobacteria*, *Firmicutes*, *Actinobacteriota,* and *Fusobacteria*, consistent with previous studies (12, 19, 25). Biomarkers in different peri-implant conditions at the genus (1, 11, 26, 27) and species levels (14) have been identified using different approaches. Here, we applied the LEfSe to identify discriminative taxa at different levels between PM and HIs and identified 37 species that were enriched in PM. Some of these taxa were classic and putative pathogens of periodontitis and have been proposed as suspected pathogens of PI, such as *F. nucleatum, P. endodontalis, P. gingivalis, T. forsythia, T. denticola, F. alocis,* and *P. micra* (20, 28, 29). Notably, among these discriminating species, we observed several *Prevotella* species (such as *P. intermedia, P. nigrescens, and P. denticola*) that have been regarded as pathogenic peri-implant microbes. However, peri-implant inflammation is a multibacterial infection, with an increasing proportion of pathogen taxa that can promote the transformation from symbiosis to dysbiosis in the bacterial community structure and behavior characteristics. Typically, the predominant bacteria at diseased peri-implant sites consist of aerobic Gram-negative rods, facultative anaerobes, and anaerobes. These bacteria are recognized for their virulence factors, which facilitate tissue invasion and destruction. *F. nucleatum* is widely known as “bridging species” that bridges early colonizers on the tooth surface with late colonizers through its adhesins (30). *P. gingivalis* interferes with the host immune system by inhibiting the early secretion of cytokines and chemokines (31). In addition, *P. gingivalis* degrades IL-8 through gingipains and prevents IL-8 transcription *via* serine phosphatase, thus impeding the function of chemokines in the immune response (32). *T. denticola* contributes to inflammatory-associated alveolar bone damage by releasing virulence factors such as periplasmic flagella and outer membrane vesicles. Previous research has demonstrated that these factors induce pro-inflammatory cytokines like IL-1β, IL-6, and TNF-α, leading to increased osteoclastogenesis through TLR2, MyD88, and Sp1 pathways. This inflammatory response results in increased RANKL expression and decreased OPG expression, promoting bone resorption and periodontal tissue damage (33). *Prevotella* species produce a variety of virulence factors, including proteases, lipopolysaccharides (LPS), hyaluronidase, and collagenase, which degrade host structural proteins such as collagen and elastin, initiating a robust inflammatory response (34). This inflammatory response activates host immune cells through TLR4 and other pathways, resulting in the release of cytokines such as TNF-α, IL-1β, and IL-6, which exacerbate tissue and bone destruction.

Similar clinical symptoms and biological features have been observed in PM and G because the tissues around dental implants and teeth can initiate a clinical inflammatory response to microbial stimulation. Earlier research found similarity in the bacterial composition of the biofilm around the dental implant and its neighboring teeth and considered the microbial flora of the natural teeth as a ‘‘reservoir’’ for the biofilms around implants (35). In this study, we found no significant difference in the alpha diversity of the submucosal microbial communities at the PM and G sites, which is in agreement with the results of a previous study (17). However, higher alpha diversity was observed in G (18). Differences in the surface roughness, free energy, and chemical composition can affect biofilm formation (36). Material characteristics can contribute to the initial events in the formation of oral biofilms and surface characteristics, such as roughness and surface-free energy, can influence the microbial composition of implants or teeth (37, 38). The differences in tissue structure and chemical composition between implant abutments and teeth can explain the variation in plaque accumulation rates between the groups and may lead to the distinction of microbial metastasis in implants. The structures of the submucosal microbial communities at the PM and G sites were significantly different, which is consistent with previous findings (15, 18). While earlier findings did not show pathogenic differences between PM and G, one study observed that the proportions of individual microbial taxa showed no differences between teeth and implants during experimental plaque accumulation; however, this was based on a DNA-DNA hybridization approach (3). The genus *Prevotella* was positively correlated with clinical inflammation in both the experimental PM and G groups (17), as in our findings, the two groups shared some of the high-abundance *Prevotella* species, with no significant differences. In agreement with our study, those on the G-associated microbiome (39–41) included species belonging to the genera *Prevotella*, *Fusobacterium*, *Tannerella*, and *Treponema*.

Whole-genome shotgun sequencing provides an opportunity to characterize the microbial community structure more precisely. Moreover, it offers insights into the functional potential of the microbiome. Of the distinguishing functional units identified by KEGG, deleterious functions involving cell motility, including bacterial chemotaxis (ko02030) and flagellar assembly (ko02040), were significantly more abundant in PM than in HIs or G. A previous study based on metatranscriptomic analyses of the PI microbiota in a periodontitis cohort presented these two findings (42). Their pathogenicity is not restricted to motility, they also participate in other microbial processes, such as host cell adherence, biofilm formation, and host cell invasion (43). Although the immunological events of peri-implant infections are qualitatively similar to periodontal ones, peri-implant inflammation usually shows a faster and more extensive progression of tissue destruction owing to differential attachment mode. The implant surface structure and abutment interface may affect microbial colonization and disease progression (44, 45), and this may be due to the differences in microbial function. Another distinct function is protein processing in the ER (ko04141). The ER is an intracellular organelle responsible for proper protein folding through the activation of several chaperone proteins. Some cellular disturbances may disrupt the ER efficiency and result in the accumulation of unfolded proteins, thereby intensifying the ER stress response. The unfolded protein response enhances the secretion of inflammatory cytokines, which influence immune responses and amplify inflammation (46). One study reported that the trait of inflamed synovitis is the overexpression of ER stress proteins (47). Prolonged ER stress can also lead to cellular dysfunction or apoptosis, thereby increasing tissue damage and intensifying symptoms of inflammatory diseases (48). Evidence of its influence on the peri-implant surrounding tissues is still not available; however, it is logical to speculate that this pathway not only plays a role in the inflammatory process of compromised dental implants but may also contribute to the accelerated tissue degradation observed in PM compared to G. Another enriched pathway in PM is pentose and glucuronate interconversions. It is a pathway that involved with the formation of D-glucuronic acid from glucose. Currently, there is limited evidence linking this pathway to inflammatory diseases. Further research is needed to investigate their potential roles in inflammation.

By contrast, reduced metabolism of several cofactors and vitamins was detected in PM. Notably, some of these compounds have anti-inflammatory effects. Folate may influence inflammatory responses *via* DNA methylation and synthesis processes (49). Lipoic acid has proved capable of abolishing the elevated myocardial inflammatory factor of interleukin (IL)-1β, IL-6, and tumor necrosis factor α (TNF-α) in rat models (50). A series of porphyrins can inhibit the production of TNF-α by directly inhibiting the activity of a non-receptor Src-family tyrosine kinase named Fyn (51). The specific roles of these cofactors and vitamins in PM remain unclear; however, the reduced metabolism of these substances indicates an active inflammatory response in PM.

We further examined the alteration in the microbial community structure and its association with disease severity. As indicated in previous studies (52–57), bacterial dysbiosis is positively correlated with more severe clinical features, such as in diabetes, obesity, Crohn’s disease, periodontitis, and PI. Certain microbial pathogens can cause the benign microbiota to become dysbiotic in emerging inflammatory diseases (58). To evaluate bacterial dysbiosis in submucosal plaques, we calculated the MDI of the PM. Inflammation in the soft tissue is a typical clinical feature of PM; therefore, the SBI (59) was used to evaluate the inflammatory status. A positive correlation between microbial dysbiosis and the clinical inflammatory status in PM is shown in Fig. 3. We also found a decreasing tendency in alpha diversity as the SBI increased; however, the correlations were not significant (Fig. S6). Meanwhile, PM samples with a lower SBI showed a more similar microbial community structure to HI samples in their beta diversity. This highlights the importance of stratifying PM of varying severities to characterize the PM microbiome. Among the most differentially abundant genera between PM and HIs, the genus *Prevotella* is positively correlated with the SBI. *Prevotella* is considered one of the early colonizers in the complex process of biofilm formation (60, 61) by promoting cell-cell adhesion and creating physical and biochemical conditions that favor later colonizers, which is necessary for stable biofilm formation (62, 63). This may indicate that a change in the relative abundance of *Prevotella* contributes to the clinical course of PM. We also performed a functional potential analysis of the submucosal plaque and noticed that positive correlations existed between the SBI and certain functions involving cell motility and protein processing. To the best of our knowledge, this is the first study to demonstrate this correlation in peri-implant submucosal plaques.

Challenges in microbiome research should not be overlooked; hence, conclusions from these microbiome studies should be interpreted cautiously. Estimation of sample size is always controversial in metagenomic studies owing to its dependence on the variance that exists among samples and high-dimensional features of metagenomic data. Therefore, statistical tests need to consider the intrinsic characteristics of microbiome data sets that do not apply to classic sample size calculation. Although it is always preferable to have a larger sample size, exhaustive investigation of microbial communities is impractical and expensive. Here, we estimated the sample size by referring to the previous studies on periodontal or peri-implant microbiota (12, 26, 64, 65). Future studies with larger sample sizes are necessary to provide insights into the features of PM from a microbial perspective. Furthermore, different sampling procedures may have led to variations in the results. Some studies sampled from the deepest probing site of an implant (14, 21), as we did, while others sampled from two, four, or six sites around the implant (3, 15, 19). Apart from the sampling sites, some studies extracted the submucosal plaque with sterile curettes (14, 15, 24, 65), whereas others used paper points (11, 12, 19, 28, 64). Different sequencing methods, such as 16S rRNA sequencing and shotgun metagenomics, result in variable sequencing depths and, therefore, may result in different outcomes. Notably, even using the same sequencing approach, there exist various methods for classifying metagenomic data and estimating taxonomic profiles. Some tools are designed to show relative sequence abundance, whereas others show relative taxonomic abundance and distinctions between them can lead to different conclusions (66). Studies on the microbiome can also have potential biases that contribute to confounding, from convenience sampling and the inclusion of heterogeneous subjects to batch effects in laboratory processes. We considered oral hygiene, systemic health, and geographic region in our study population; however, five of the subjects in the PM group had periodontitis, and four were known smokers. These may confound the peri-implant submucosal microbiome with different periodontal health and smoking habits (24, 25). Future studies with adequate control for confounding factors are recommended to confirm our findings. The batch effect is a systematic, non-biological difference between batches that is common in genomics experiments and may result in misleading conclusions. Sound experimental designs and statistical analysis methods are required to minimize its impact. The allocation of samples to batches should be considered in future studies to reduce confounding or correlations between batches and the biological variables of interest. As 5%–45% of inter-individual variation can be explained by genetics (67), host-associated microbial communities are influenced by both host genetics and environmental factors. Besides, gene polymorphisms and differentially expressed genes may also contribute to differences in inflammation progression (68, 69). Therefore, further studies are necessary to explore the interplay between genetic factors and environmental influences in peri-implant diseases. The strength of our study is that we included an intra-subject control group when investigating the differences between PM and G. Future studies on the interactions between the host and microbiome are necessary to better understand PM pathogenesis.

## MATERIALS AND METHODS

### Subject recruitment and sampling

This study was conducted in accordance with the 1964 Helsinki Declaration and its later amendments or comparable ethical standards and was approved by the Ethics Committee at Shanghai Fifth People’s Hospital affiliated with Fudan University [(2019) 101]. It also conformed to strengthening the reporting of observational studies in epidemiology (STROBE) guidelines for observational studies (70). Informed consent was obtained from all participants enrolled in this study.

Study participants were recruited from the Department of Stomatology of Shanghai Fifth People’s Hospital between March 2023 and September 2023. Participants had to be at least 18 years old, had to be systemically healthy, and had to have not received any antibiotics or anti-inflammatory drugs within 3 months before the examination. We excluded participants who were fully edentulous, pregnant, or lactating, had used any medication, or had received treatment known to affect periodontal health in the previous 2 weeks, were receiving steroid medications, or had a heavy smoking habit (>20 cigarettes/day). All participants had at least one implant restored with crowns or prostheses for at least 1 year.

The diagnostic criteria were based on the consensus report of Workgroup 4 of the 2017 World Workshop on the Classification of Periodontal and Peri-Implant Diseases and Conditions (6). For the PM group, if a patient had multiple implants affected by PM, the implant exhibiting the most severe bleeding on probing was selected for clinical examination and sample collection. The selection criteria for the HI group required that all dental implants in one single patient be completely healthy, with each implant in patients having multiple implants needing to be free of inflammatory symptoms; in this case, an HI was randomly selected from the eligible implants in a single participant. Gingivitis (G) was defined as the presence of bleeding on gentle probing at one or more sites, accompanied by clinical signs of inflammation such as redness and swelling of the gingiva, without attachment loss. Study participants with PM were further divided according to their periodontal conditions, and participants with G in the tooth adjacent to the diseased implant were selected for subgingival plaque collection on this tooth.

Before the survey, the two authors performed a standard consistency test for clinical parameter measurements. Two examiners independently performed clinical examinations of the sampling sites. A kappa coefficient value of ≥0.90 was used, for which intra- and inter-examiner reproducibility was determined, indicating high consistency.

All participants then underwent a full-mouth periodontal examination, where each implant was probed at six sites, and the maximum probing depth was recorded as the pocket probing depth (PPD). Clinical parameters such as the plaque index (PLI), the sulcus bleeding index (SBI), suppuration (SUP), and marginal bone loss (MBL) on radiography were measured at each sampling site. Information on the participants’ oral hygiene practices and smoking habits was also recorded.

The submucosal/subgingival plaques were collected from the site with the greatest probe depth. Furthermore, the sampling site was isolated and air-dried, and the supragingival plaque was removed using sterile cotton pellets. The submucosal/subgingival plaque samples were collected using three sterile paper points (30#) inserted into the base of the pocket and maintained for 30 s. The samples were immediately placed in labeled Eppendorf tubes (Eppendorf, Hamburg, Germany) containing sterile phosphate-buffered saline solution (Thermo Fisher Scientific, Waltham, MA, USA) and stored at −80°C for transportation to the laboratory for the subsequent DNA extraction.

### Metagenome DNA extraction and sequencing data collection

Total microbial genomic DNA samples were extracted using the Mag-Bind Soil DNA kit (Omega Bio-tek, Norcross, GA, USA) (M5635-02) according to the manufacturer’s protocol. The isolated DNA was then stored at −20°C. The prepared sample buffer was subjected to laboratory-controlled extraction to identify the potential contaminants. Furthermore, the quantity and quality of the extracted DNA were measured using a Qubit 4 Fluorometer (Invitrogen, USA) and agarose gel electrophoresis, respectively. The extracted microbial DNA was processed using the Illumina TruSeq Nano DNA LT Library Preparation Kit to construct metagenome shotgun sequencing libraries with an insert size of 400 bp. We sequenced each library using the Illumina NovaSeq platform (Illumina, USA) with the PE150 strategy at Personal Biotechnology Co., Ltd. (Shanghai, China). Raw sequencing reads were processed to obtain quality-filtered reads for further analysis. We removed sequencing adapters from the sequencing reads using Cutadapt (version 1.2.1) (71). Thereafter, low-quality reads were trimmed using the sliding window algorithm in fastp (72), and the reads were aligned to the human host genome using BMTagger to remove host contamination (73).

### Taxonomic composition and microbial community structure analysis

After obtaining quality-filtered reads, the taxonomic classification of the metagenomic sequencing reads from each sample was performed using Kraken2 (74) against a RefSeq-derived database. We removed reads assigned to Metazoans or Viridiplantae from the downstream analysis, and Megahit (version 1.1.2) (75) was used to assemble each sample using meta-large preset parameters. Furthermore, generated contigs (longer than 300 bp) were pooled together and clustered using mmseqs2 (76) with the “easy-linclust” mode, setting the sequence identity threshold to 0.95 and the covered residue of the shorter contig to 90%. The lowest common ancestor taxonomy of the non-redundant contigs was obtained by aligning them against the NCBI-nt database by mmseqs2 (76) with the “taxonomy” mode and dropped contigs assigned to Viridiplantae or Metazoa in the following analysis.

Alpha diversity analysis was performed using Shannon and Simpson indices in the base R package (77). We performed a beta diversity analysis of taxonomic profiles based on the Bray-Curtis distance to describe the compositional distribution of the samples through a two-dimensional ordination map in the Vegan R package (78) and visualized it using nonmetric multidimensional scaling (NMDS). The linear discriminant analysis effect size (LEfSe) was used to identify statistically significant biomarkers among the groups (79). A size-effect threshold of 3.0 on the logarithmic linear discriminant analysis (LDA) score was used to identify discriminating taxa from the phylum to the species level.

We plotted heatmaps using heatmap tools on a free online platform for data analysis, GenesCloud (https://www.genescloud.cn). This tool was developed using the pheatmap package (version 1.0.8) in R, and the data were normalized to z-scores. This package uses the popular clustering distances and methods implemented in the Dist and Hclust functions in R. In this study, we adopted the Euclidean clustering distance and complete clustering methods.

### Functional profile analyses

We annotated the functionality of the non-redundant genes using mmseqs2 (76) with the “search” mode against the Kyoto Encyclopedia of Genes and Genomes (KEGG) database (80) to obtain the KEGG orthologies (KOs). Beta diversity analysis was performed to investigate the functional variation in microbial communities across samples using Bray-Curtis distance metrics (81) and was visualized *via* NMDS. Based on the functional profiles of non-redundant genes, the LEfSe was used to detect differentially abundant functions across the groups (79). Pathways with a relative abundance of >0.05% in samples from at least two subjects were chosen for analysis, and the LDA score was set to a threshold of 3 in the functional analysis.

### Correlation analyses

The microbial dysbiosis index (MDI) was calculated to evaluate microbiome dysbiosis (54). The correlations between the SBI and MDI with the discriminating taxa and functional pathways in the PM plaques were calculated using Pearson correlation coefficient analysis.

### Statistical analyses

Mann-Whitney U test and chi-square analysis were used as appropriate statistical tests for clinical and demographic parameters. The Kruskal–Wallis rank sum test was used to compare significant differences in alpha diversity among the different groups. We set *P* < 0.05 as the threshold for statistical significance. Statistical significance among the groups in terms of beta diversity was confirmed using permutational multivariate analysis of variance (PERMANOVA) (82), and comparisons between the two groups were conducted using analysis of similarities (ANOSIM) (83). All statistical analyses were performed using IBM SPSS Statistics (version 26.0) and R (version 4.1.3).

## Data Availability

Raw data are deposited in the Sequence Read Archive (SRA, accession number: PRJNA1064442), code used to analyze the data is available on GitHub. Other reagents and protocols are available upon request.

## SUPPLEMENTAL MATERIAL

Fig. S1 msphere.00059-24-s0001.pdf The species accumulation curve of each study group.

Fig. S2 msphere.00059-24-s0002.pdf The overall taxonomic composition of the three study groups at phylum level.

Fig. S3 msphere.00059-24-s0003.pdf Biomarkers indicated by LEfSe analysis.

Fig. S4 msphere.00059-24-s0004.pdf Composition of the top 10 species in each study group.

Fig. S5 msphere.00059-24-s0005.pdf Biomarkers in functional potential.

Table S1 msphere.00059-24-s0006.xlsx Clinical and demographic information of each subject.

ASM does not own the copyrights to Supplemental Material that may be linked to, or accessed through, an article. The authors have granted ASM a non-exclusive, world-wide license to publish the Supplemental Material files. Please contact the corresponding author directly for reuse.
