# Supplementary figures and images for: Microbiological and functional traits of peri-implant mucositis and correlation with disease severity
Source: mSphere. 2024 Jul 9;9(7):e00059-24. doi: 10.1128/msphere.00059-24 (PMC11287996; doi:10.1128/msphere.00059-24)

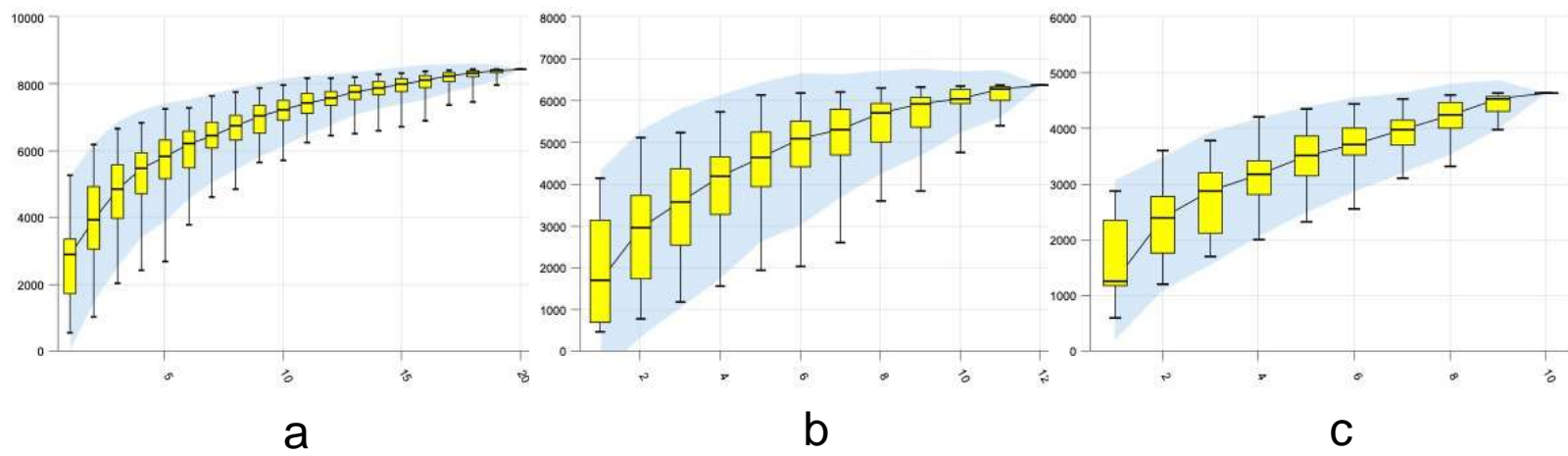

Fig.S1 The species accumulation curve of each study group: PM (a), HI (b), G (c).

Supplement: Fig. S1 — The species accumulation curve of each study group. [file msphere.00059-24-s0001.pdf]

a

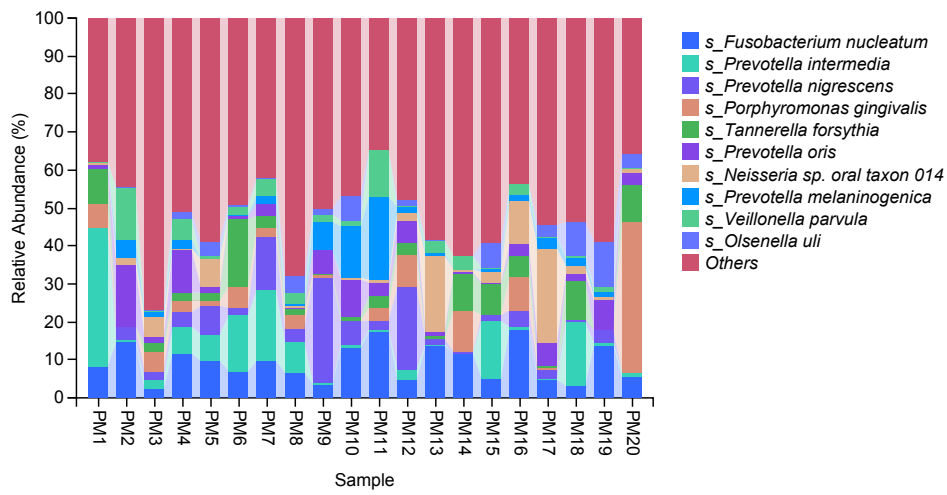

b

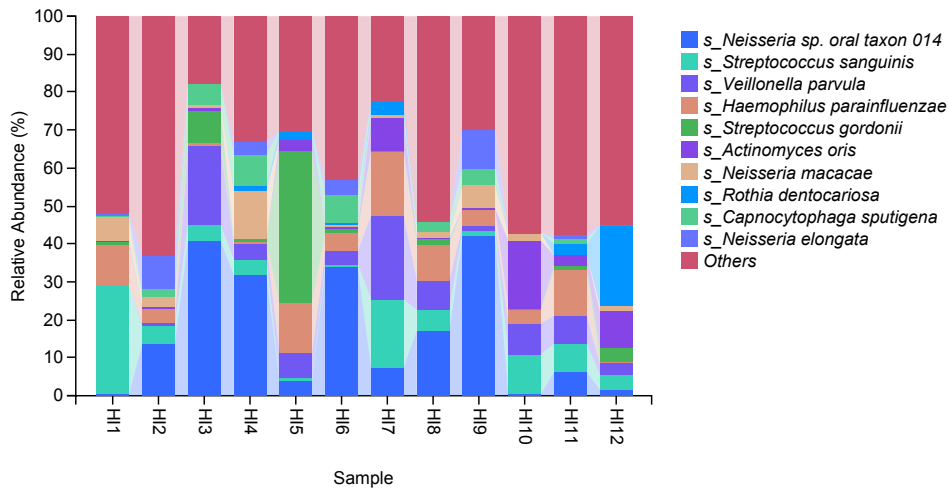

c

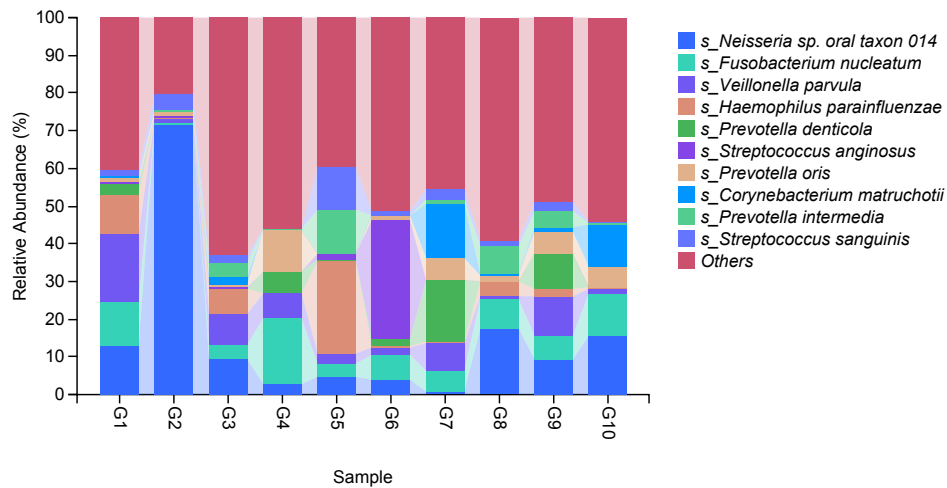

Fig.S4 Composition of the top 10 species in each study group: PM (a), HI (b), G (c).

Supplement: Fig. S4 — Composition of the top 10 species in each study group. [file msphere.00059-24-s0004.pdf]
