# Supplementary material for: Microbiological and functional traits of peri-implant mucositis and correlation with disease severity
Source: mSphere. 2024 Jul 9;9(7):e00059-24. doi: 10.1128/msphere.00059-24 (PMC11287996; doi:10.1128/msphere.00059-24)
Supplement: Fig. S2 — The overall taxonomic composition of the three study groups at phylum level. [file msphere.00059-24-s0002.pdf]

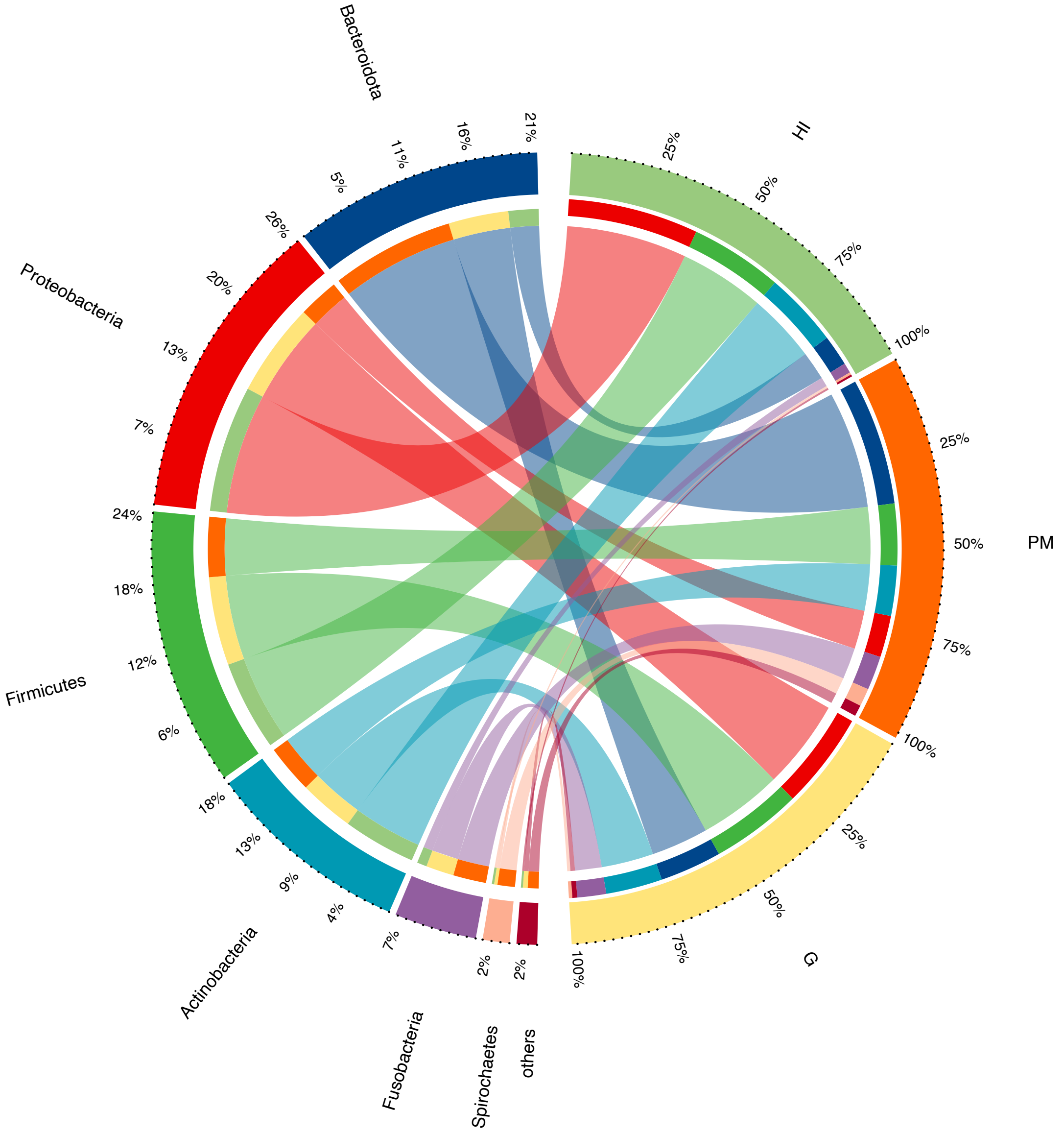

Fig.S2 The overall taxonomic composition of the three study groups and the correspondence between the taxa and sample groups at the phylum level shows different predominant taxa across groups.
