# Supplementary material for: Microbiological and functional traits of peri-implant mucositis and correlation with disease severity
Source: mSphere. 2024 Jul 9;9(7):e00059-24. doi: 10.1128/msphere.00059-24 (PMC11287996; doi:10.1128/msphere.00059-24)
Supplement: Fig. S3 — Biomarkers indicated by LEfSe analysis. [file msphere.00059-24-s0003.pdf]

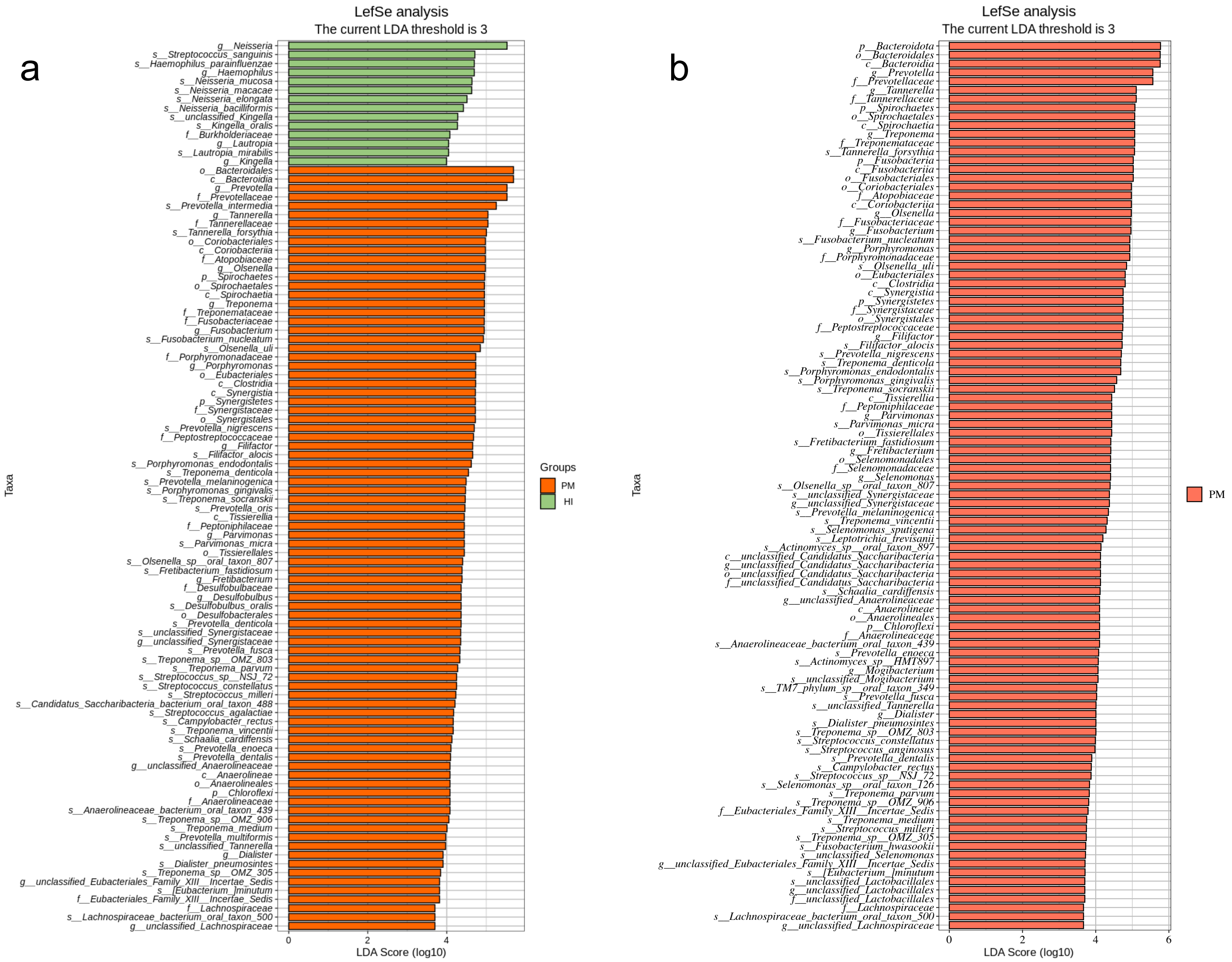

Fig.S3 (a) Biomarkers indicated by LEfSe analysis between the PM and HI groups. Only taxa with LDA values ⩾3.0 are shown. (b) Biomarkers indicated by LEfSe analysis between the PM and G groups. Only taxa with LDA values ⩾3.0 are shown.
